# Supplementary material for: The Evolution and Characterization of the RNA Interference Pathways in Lophotrochozoa
Source: Genome Biol Evol. 2024 May 7;16(5):evae098. doi: 10.1093/gbe/evae098 (PMC11114477; doi:10.1093/gbe/evae098)
Supplement: evae098_Supplementary_Data [file evae098_supplementary_data.zip › Supplementary_materials.zip!/selected_miRNAs/NoGe_selected_miRNAs/mirdeep_pdf_files/NMRB01002613.1_Notospermus_geniculatus_strain_Ushimado_scaffold2613,_whole_genome_shotgun_sequence_39530.pdf]

| | | |
|---|---|---|
| Provisional ID | : | NMRB01002613.1_Notospermus_geniculatus_strain_Ushimado_scaffold2613,_whole_genome_shotgun_sequence_39530 |
| Score total | : | 37806.6 |
| Score for star read(s) | : | 3.9 |
| Score for read counts | : | 37798.1 |
| Score for mfe | : | 1.6 |
| Score for randfold | : | 0 |
| Score for cons. seed | : | 3 |
| Total read count | : | 74151 |
| Mature read count | : | 74142 |
| Loop read count | : | 0 |
| Star read count | : | 9 |

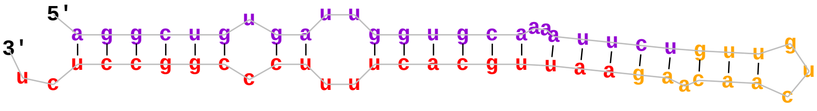


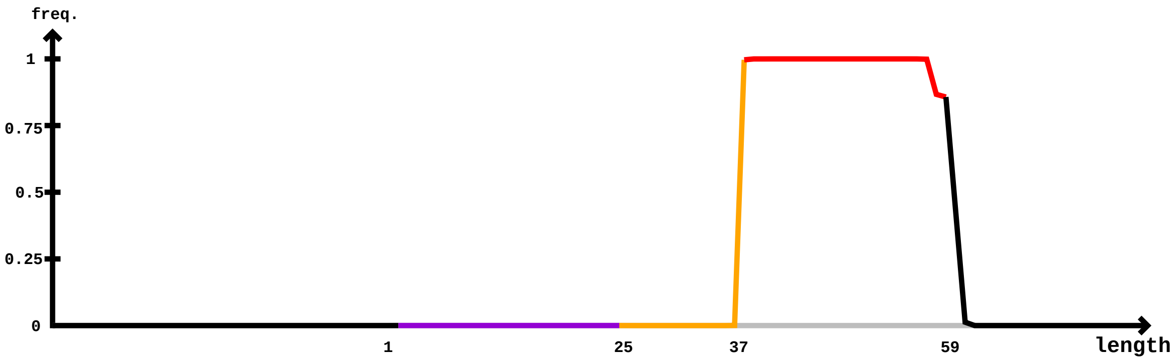


Star Mature

```
5'- accccaaacccuggaucucccggcugcuuagcaaaggcugugauugguggcaaaauucuguugucaacaagaauugcacuuuucccggccucugugcuguggccaguucuucc  -3'   obs
    accccaaacccuggaucucccggcugcuuagcaaaggcugugauugguggcaaaauucuguugucaacaagaauugcacuuuucccggccucugugcuguggccaguucuucc         exp
    ...(((.....)))........((((((...(((.((((((.((..((((((..((((((((...))).)))))))))))..)).)))))).)))...))))))..........   reads   mm   sample
    ................................aaaggcugugauugguggcaaaauu........................................................   3       0    Fm1
    ..................................aggcugugauugguggcaaaauucu......................................................   3       0    Fm1
    ......................................................................Caauugcacuuuucccggccucu....................   2       1    Fm1
    .......................................................................aauugcacuCuucccggc........................   1       1    Fm1
    .......................................................................aauugcacuuuucccggcc.......................   4       0    Fm1
    .......................................................................aauugcacuuuucccggAcu......................   23      1    Fm1
    .......................................................................aauugcacuuuucccggccC......................   1       1    Fm1
    .......................................................................aauugcacCuuucccggccu......................   1       1    Fm1
    .......................................................................Uauugcacuuuucccggccu......................   1       1    Fm1
    .......................................................................aauugcacuCuucccggccu......................   1       1    Fm1
    .......................................................................aauugcacuuuucccgUccu......................   3       1    Fm1
    .......................................................................aauugcacuuuucUcggccu......................   2       1    Fm1
    .......................................................................aauugcCcuuuucccggccu......................   1       1    Fm1
    .......................................................................aauugcacuuuucccggGcu......................   11      1    Fm1
    .......................................................................aauugcacuuAucccggccu......................   6       1    Fm1
    .......................................................................aCuugcacuuuucccggccu......................   1       1    Fm1
    .......................................................................aauugcacuuuucccgAccu......................   3       1    Fm1
    .......................................................................aauugcacuuuucccggccu......................   2117    0    Fm1
    .......................................................................aauugcacuuuucccggUcu......................   5       1    Fm1
    .......................................................................aauugcacuuuucccgCccu......................   7       1    Fm1
    .......................................................................aauugcacuuuucccggccA......................   6       1    Fm1
    .......................................................................aauugNacuuuucccggccu......................   1       1    Fm1
    .......................................................................aauugcacuuuucccggccG......................   92      1    Fm1
    .......................................................................aauugcacuuuucccAgccuc.....................   1       1    Fm1
    .......................................................................aauugcacuuuuUccggccuc.....................   1       1    Fm1
    .......................................................................aauugcacuuuucccggccuA.....................   5       1    Fm1
    .......................................................................aauugcacuuuucccggccuc.....................   114     0    Fm1
    .......................................................................aauugcacuuuucccggccuU.....................   19      1    Fm1
    .......................................................................Uauugcacuuuucccggccuc.....................   1       1    Fm1
    .......................................................................aauugcacuuuucccgUccuc.....................   1       1    Fm1
    .......................................................................aauugcacuuuucccgCccuc.....................   1       1    Fm1
    .......................................................................aauAgcacuuuucccggccucu....................   2       1    Fm1
    .......................................................................aauugcGcuuuucccggccucu....................   1       1    Fm1
```

```
                                  Star                              Mature
accccaaacccuggaucucccggcugcuuagcaaaggcugugauugguggcaaaauucuguugucaacaagaauugcacuuuucccggccucugugcuguggccaguucuucc
.......................................................................aauugcacuuAucccggccucu....................    12     1    Fm1
.......................................................................aauugcacuuuucccggccucu....................    8214   0    Fm1
.......................................................................aauugcacuuuucccggccuAu....................    1      1    Fm1
.......................................................................aauugcacuGuucccggccucu....................    3      1    Fm1
.......................................................................Cauugcacuuuucccggccucu....................    1      1    Fm1
.......................................................................aCuugcacuuuucccggccucu....................    3      1    Fm1
.......................................................................aauugcacuuuucccggccucA....................    11     1    Fm1
.......................................................................aauugcacuuuucccgCccucu....................    16     1    Fm1
.......................................................................aGuugcacuuuucccggccucu....................    1      1    Fm1
.......................................................................aauugcacuuuuUccggccucu....................    19     1    Fm1
.......................................................................aauugcacuuuucccggccuGu....................    7      1    Fm1
.......................................................................aauugcacuuuucccgAccucu....................    19     1    Fm1
.......................................................................aauugcacCuuucccggccucu....................    8      1    Fm1
.......................................................................aauugcacuuuuAccggccucu....................    2      1    Fm1
.......................................................................aauugcNcuuuucccggccucu....................    1      1    Fm1
.......................................................................aauugcacuuuucccggUcucu....................    52     1    Fm1
.......................................................................aauugcacGuuucccggccucu....................    2      1    Fm1
.......................................................................aauugcacuuuucccggGcucu....................    29     1    Fm1
.......................................................................aauugcUcuuuucccggccucu....................    2      1    Fm1
.......................................................................aauCgcacuuuucccggccucu....................    3      1    Fm1
.......................................................................aauuCcacuuuucccggccucu....................    1      1    Fm1
.......................................................................aauugcacAuuucccggccucu....................    1      1    Fm1
.......................................................................aaGugcacuuuucccggccucu....................    2      1    Fm1
.......................................................................aauugcacuuuuccAggccucu....................    2      1    Fm1
.......................................................................aauuAcacuuuucccggccucu....................    1      1    Fm1
.......................................................................aauugcacuuuucccggccGcu....................    1      1    Fm1
.......................................................................aauugcacuuuucccggccucC....................    12     1    Fm1
.......................................................................aauugcacuuuucccggAcucu....................    93     1    Fm1
.......................................................................aauugcacuuGucccggccucu....................    12     1    Fm1
.......................................................................aauugcacuuuucccAgccucu....................    1      1    Fm1
.......................................................................aaCugcacuuuucccggccucu....................    1      1    Fm1
.......................................................................aauugNacuuuucccggccucu....................    5      1    Fm1
.......................................................................aauugcacuuuucccUgccucu....................    1      1    Fm1
.......................................................................aauugcaUuuuucccggccucu....................    2      1    Fm1
.......................................................................aauugcacuuuucccgUccucu....................    21     1    Fm1
.......................................................................aauugcacuuuucUcggccucu....................    4      1    Fm1
.......................................................................aUuugcacuuuucccggccucu....................    4      1    Fm1
.......................................................................aaAugcacuuuucccggccucu....................    2      1    Fm1
.......................................................................aauugcacuuuucccggccucG....................    116    1    Fm1
.......................................................................Uauugcacuuuucccggccucu....................    15     1    Fm1
.......................................................................aauugcacuuuucccggccuUu....................    14     1    Fm1
.......................................................................Gauugcacuuuucccggccucu....................    2      1    Fm1
.......................................................................Nauugcacuuuucccggccucu....................    2      1    Fm1
.......................................................................aauugcacuuCucccggccucu....................    9      1    Fm1
.......................................................................aauugcacuuuuccUggccucu....................    3      1    Fm1
.......................................................................aauugcacuuuucAcggccucu....................    2      1    Fm1
.......................................................................aauugcacuuuucccggcUucu....................    5      1    Fm1
.......................................................................aauugcacuuuucccggccucuC...................    7      1    Fm1
.......................................................................aauugcacuuuucccggccucuA...................    31     1    Fm1
.......................................................................aauugcacuuuucccggccucuU...................    247    1    Fm1
.......................................................................aauugcacuuuucccggccucuAu..................    28     1    Fm1
.......................................................................aauugcacuuuucccggccucuUu..................    126    1    Fm1
.......................................................................aauugcacuuuucccggccucugu..................    1      0    Fm1
........................................................................auugcacuuuucccggccuGu....................    1      1    Fm1
........................................................................auugcacuuuucccggcUucu....................    1      1    Fm1
........................................................................auugcacuuuucccggcGucu....................    1      1    Fm1
........................................................................auugcacuuuucccggccucu....................    22     0    Fm1
........................................................................auugcacuuuCcccggccucu....................    1      1    Fm1
........................................................................auugcacuuuucccggccucug...................    1      0    Fm1
........................................................................auugcacuuuucccggccucuA...................    2      1    Fm1
........................................................................auugcacuuuucccggccucuU...................    1      1    Fm1
........................................................................auugcacuuuucccggccucugu..................    9      0    Fm1
........................................................................auugcacuuuucccggccucugG..................    1      1    Fm1
........................................................................auugcacuuuucccggccucuAu..................    6      1    Fm1
.........................................................................uugcacuuuucccggccucu....................    2      0    Fm1

.......................................................................aauugcacuuuucccggAcu......................    13     1    Fm2
.......................................................................aauugcacuuuucccggUcu......................    3      1    Fm2
.......................................................................aauugcacuuuucccggcGu......................    1      1    Fm2
.......................................................................aauugcacuuuucccggccG......................    39     1    Fm2
```

```
                                  Star                                Mature
accccaaacccuggaucucccggcugcuuagcaaaggcugugauuggugcaaaauucuguugucaacaagaauugcacuuuucccggccucugugcugguggccaguucuucc
......................................................................aauugcacuuuucccggGcu.......................   2      1     Fm2
......................................................................aauugcacuuuucccgAccu.......................   3      1     Fm2
......................................................................aauugcacGuuucccggccu.......................   1      1     Fm2
......................................................................aauugcacuuAucccggccu.......................   1      1     Fm2
......................................................................Uauugcacuuuucccggccu.......................   1      1     Fm2
......................................................................aauuUcacuuuucccggccu.......................   1      1     Fm2
......................................................................aUuugcacuuuucccggccu.......................   1      1     Fm2
......................................................................aauugcacuuuucUcggccu.......................   1      1     Fm2
......................................................................aauugcacuuuucccggccu.......................   764    0     Fm2
......................................................................aauugcacuuuucccgCccu.......................   1      1     Fm2
......................................................................aauugcacuuuuccUggccu.......................   2      1     Fm2
......................................................................aaGugcacuuuucccggccu.......................   1      1     Fm2
......................................................................Gauugcacuuuucccggccu.......................   1      1     Fm2
......................................................................aauugcacuuCucccggccu.......................   1      1     Fm2
......................................................................aauugcacuuuucccgUccu.......................   1      1     Fm2
......................................................................aauugcacuuuucccggAcuc......................   1      1     Fm2
......................................................................aauugcacuuuucccggccuU......................   18     1     Fm2
......................................................................aauugcacuuuucccggccuc......................   54     0     Fm2
......................................................................aauugcacuuuucccggccCcu.....................   1      1     Fm2
......................................................................aauugcacuuuucccgAccucu.....................   11     1     Fm2
......................................................................aauuAcacuuuucccggccucu.....................   1      1     Fm2
......................................................................aauugcacCuuucccggccucu.....................   4      1     Fm2
......................................................................aauugcacuuAucccggccucu.....................   7      1     Fm2
......................................................................aauugUacuuuucccggccucu.....................   1      1     Fm2
......................................................................aauuUcacuuuucccggccucu.....................   1      1     Fm2
......................................................................aauugcacuuuucccAgccucu.....................   2      1     Fm2
......................................................................aauugcacuuuucccggUcucu.....................   33     1     Fm2
......................................................................Gauugcacuuuucccggccucu.....................   2      1     Fm2
......................................................................aauuCcacuuuucccggccucu.....................   1      1     Fm2
......................................................................aauugcacuuuuUccggccucu.....................   1      1     Fm2
......................................................................Cauugcacuuuucccggccucu.....................   2      1     Fm2
......................................................................aauugcaUuuuucccggccucu.....................   1      1     Fm2
......................................................................aaCugcacuuuucccggccucu.....................   1      1     Fm2
......................................................................aauugcacuuuucccggccucC.....................   8      1     Fm2
......................................................................aauugcacuuuucccggccucA.....................   2      1     Fm2
......................................................................aauugcacuuuucccgUccucu.....................   13     1     Fm2
......................................................................aauAgcacuuuucccggccucu.....................   1      1     Fm2
......................................................................Uauugcacuuuucccggccucu.....................   10     1     Fm2
......................................................................aauugcacuuuuccUggccucu.....................   2      1     Fm2
......................................................................aauugcacuuuucccggGcucu.....................   21     1     Fm2
......................................................................aCuugcacuuuucccggccucu.....................   2      1     Fm2
......................................................................aauugcacuuuucAcggccucu.....................   6      1     Fm2
......................................................................Nauugcacuuuucccggccucu.....................   1      1     Fm2
......................................................................aauugcacuuuuccAggccucu.....................   2      1     Fm2
......................................................................aauugcacuuuucccggcAucu.....................   1      1     Fm2
......................................................................aauugcacuuCucccggccucu.....................   6      1     Fm2
......................................................................aauugcacuuuucccggccucG.....................   111    1     Fm2
......................................................................aauugcacuuGucccggccucu.....................   3      1     Fm2
......................................................................aauugcacuuuucccggccucu.....................   5418   0     Fm2
......................................................................aauugcGcuuuucccggccucu.....................   2      1     Fm2
......................................................................aauugcacAuuucccggccucu.....................   1      1     Fm2
......................................................................aauugcacuuuucccggccuUu.....................   2      1     Fm2
......................................................................aauugcacuuuuccGggccucu.....................   1      1     Fm2
......................................................................aauugcacuuuucccgCccucu.....................   14     1     Fm2
......................................................................aauugGacuuuucccggccucu.....................   2      1     Fm2
......................................................................aauugcacuuuucccggcUucu.....................   1      1     Fm2
......................................................................aauugcacuuuucUcggccucu.....................   5      1     Fm2
......................................................................aauugcacuuuucccggAcucu.....................   46     1     Fm2
......................................................................aauugcacuuuucccggccucuU....................   279    1     Fm2
......................................................................aauugcacuuuucccggccucug....................   3      0     Fm2
......................................................................aauugcacuuuucccggccucuA....................   76     1     Fm2
......................................................................aauugcacuuuucccggccucuUu...................   116    1     Fm2
......................................................................aauugcacuuuucccggccucuAu...................   30     1     Fm2
.......................................................................auugcacuuuucccggccucu.....................   5      0     Fm2
.......................................................................auugcacuuuucccggccucuA....................   2      1     Fm2

.....................................................................gaauugcacuuuucccggccucu.....................   1      0     Mm1
......................................................................aauugcacuCuucccggcc........................   1      1     Mm1
......................................................................aauugcacuuuucccgUccu.......................   1      1     Mm1
......................................................................aauugcacuuAucccggccu.......................   2      1     Mm1
```

Star Mature

| | | | |
|---|---|---|---|
| accccaaacccuggaucucccggcugcuuagcaaaggcugugauuggugcaaaauucuguugucaacaagaauugcacuuuucccggccucugugcuguggccaguucuucc | | | |
| ..........................................................................aauugcacuuuuccUggccu...................... | 1 | 1 | Mm1 |
| ......................................................................aauugcacuuuucccgCccu...................... | 1 | 1 | Mm1 |
| ......................................................................aauugcacuuuucccggccu...................... | 296 | 0 | Mm1 |
| ......................................................................aUuugcacuuuucccggccu...................... | 1 | 1 | Mm1 |
| ......................................................................Uauugcacuuuucccggccu...................... | 1 | 1 | Mm1 |
| ......................................................................aauugcacuCuucccggccu...................... | 4 | 1 | Mm1 |
| ......................................................................aauugcacuuuucccggccG...................... | 1 | 1 | Mm1 |
| ......................................................................aauugcacuuuucccggAcu...................... | 2 | 1 | Mm1 |
| ......................................................................aauugcacuuuucccggccuc..................... | 2 | 0 | Mm1 |
| ......................................................................aauugcacuuuucccgCccuc..................... | 1 | 1 | Mm1 |
| ......................................................................aauugcacuuuucccggAcucu.................... | 3 | 1 | Mm1 |
| ......................................................................Uauugcacuuuucccggccucu.................... | 3 | 1 | Mm1 |
| ......................................................................aauugcacuuuucccggccucu.................... | 812 | 0 | Mm1 |
| ......................................................................aauugcacuuGucccggccucu.................... | 1 | 1 | Mm1 |
| ......................................................................aauugcacuuuucccgUccucu.................... | 2 | 1 | Mm1 |
| ......................................................................aauugcacuuuucUcggccucu.................... | 1 | 1 | Mm1 |
| ......................................................................aauugcacuuAucccggccucu.................... | 2 | 1 | Mm1 |
| ......................................................................aauugcacuuuucccggcUucu.................... | 1 | 1 | Mm1 |
| ......................................................................aauugcacuuCucccggccucu.................... | 1 | 1 | Mm1 |
| ......................................................................aaAugcacuuuucccggccucu.................... | 1 | 1 | Mm1 |
| ......................................................................aauugUacuuuucccggccucu.................... | 2 | 1 | Mm1 |
| ......................................................................aauCgcacuuuucccggccucu.................... | 2 | 1 | Mm1 |
| ......................................................................aauugcacuuuucccggUcucu.................... | 5 | 1 | Mm1 |
| ......................................................................aauugcacuuuucccggGcucu.................... | 1 | 1 | Mm1 |
| ......................................................................aauugcacuuuucccgAccucu.................... | 4 | 1 | Mm1 |
| ......................................................................aauugcacuuuCcccggccucu.................... | 1 | 1 | Mm1 |
| ......................................................................aauugcacuAuucccggccucu.................... | 1 | 1 | Mm1 |
| ......................................................................aauugcacuuuucccggccucA.................... | 4 | 1 | Mm1 |
| ......................................................................aauugcacuuuucccggccuGu.................... | 24 | 1 | Mm1 |
| ......................................................................aauugcacuuuucccggccucuU................... | 22 | 1 | Mm1 |
| ......................................................................aauugcacuuuucccggccucuA................... | 10 | 1 | Mm1 |
| ......................................................................aauugcacuuuucccggccucuAu.................. | 1 | 1 | Mm1 |
| ......................................................................aauugcacuuuucccggccucuUu.................. | 8 | 1 | Mm1 |
| .......................................................................auugcacuuuucccggccu...................... | 1 | 0 | Mm1 |
| .......................................................................auugcacuuuucccggccuGu.................... | 1 | 1 | Mm1 |
| .......................................................................auugcacuuuucccggccucu.................... | 8 | 0 | Mm1 |
| .......................................................................auugcacuuuucccggccucG.................... | 1 | 1 | Mm1 |
| ..................................aggcugugauuggugcaaaauucu...................................................... | 2 | 0 | Mm2 |
| .....................................................................gaauugcacuuuucccggccu...................... | 5 | 0 | Mm2 |
| .....................................................................Caauugcacuuuucccggccu...................... | 4 | 1 | Mm2 |
| .....................................................................gaauugcacuuuucccggccucA.................... | 1 | 1 | Mm2 |
| .....................................................................Caauugcacuuuucccggccucu.................... | 9 | 1 | Mm2 |
| .....................................................................gaauugcacuuuucccggccucu.................... | 1 | 0 | Mm2 |
| ......................................................................aauugcacuuuucccggc........................ | 1 | 0 | Mm2 |
| ......................................................................aauugcacuuuucccggcc....................... | 28 | 0 | Mm2 |
| ......................................................................aauugcacuGuucccggccu...................... | 1 | 1 | Mm2 |
| ......................................................................aauugcUcuuuucccggccu...................... | 2 | 1 | Mm2 |
| ......................................................................aauugcacuuuucccggccu...................... | 3571 | 0 | Mm2 |
| ......................................................................aauugcacuuuucccgAccu...................... | 8 | 1 | Mm2 |
| ......................................................................aCuugcacuuuucccggccu...................... | 1 | 1 | Mm2 |
| ......................................................................aauugcacuuuucccgCccu...................... | 11 | 1 | Mm2 |
| ......................................................................aauugcaUuuuucccggccu...................... | 1 | 1 | Mm2 |
| ......................................................................aGuugcacuuuucccggccu...................... | 1 | 1 | Mm2 |
| ......................................................................aauugcacuuAucccggccu...................... | 4 | 1 | Mm2 |
| ......................................................................aauGgcacuuuucccggccu...................... | 1 | 1 | Mm2 |
| ......................................................................aauuCcacuuuucccggccu...................... | 1 | 1 | Mm2 |
| ......................................................................aauugcacuuuucccggcUu...................... | 1 | 1 | Mm2 |
| ......................................................................aauugcacuuuucccggccC...................... | 3 | 1 | Mm2 |
| ......................................................................aauugcacuuuucccggAcu...................... | 43 | 1 | Mm2 |
| ......................................................................Uauugcacuuuucccggccu...................... | 4 | 1 | Mm2 |
| ......................................................................Gauugcacuuuucccggccu...................... | 1 | 1 | Mm2 |
| ......................................................................aauugcacCuuucccggccu...................... | 1 | 1 | Mm2 |
| ......................................................................aauugcacuuuuccUggccu...................... | 1 | 1 | Mm2 |
| ......................................................................aauugcacuuuucccggGcu...................... | 12 | 1 | Mm2 |
| ......................................................................aauugcCcuuuucccggccu...................... | 1 | 1 | Mm2 |
| ......................................................................aauugcacuuuucccggccA...................... | 16 | 1 | Mm2 |
| ......................................................................aauuUcacuuuucccggccu...................... | 3 | 1 | Mm2 |
| ......................................................................aauugcacuuuucccggccG...................... | 104 | 1 | Mm2 |
| ......................................................................aauCgcacuuuucccggccu...................... | 1 | 1 | Mm2 |

Star                Mature

```
accccaaacccuggaucucccggcugcuuagcaaaggcugugauugggugcaaaauucuguugucaacaagaauugcacuuuucccggccucugugcuguggccaguucuucc
.......................................................................aauugcacuuuAcccggccu......................    1      1    Mm2
.......................................................................aauugcGcuuuucccggccu......................    1      1    Mm2
.......................................................................aauugcacuuuucccggUcu......................    14     1    Mm2
.......................................................................aauugcacuuuCcccggccu......................    1      1    Mm2
.......................................................................aauugcacuuuucccgUccu......................    12     1    Mm2
.......................................................................aauugcacuuCucccggccu......................    7      1    Mm2
.......................................................................aauugcacuuuuUccggccu......................    1      1    Mm2
.......................................................................aauugNacuuuucccggccu......................    2      1    Mm2
.......................................................................aauugUacuuuucccggccu......................    3      1    Mm2
.......................................................................aauAgcacuuuucccggccu......................    2      1    Mm2
.......................................................................aauugcacuCuucccggccu......................    3      1    Mm2
.......................................................................aauugcacuuuucccggccuc.....................    305    0    Mm2
.......................................................................aauugcacuuuucccgCccuc.....................    1      1    Mm2
.......................................................................aauugcacuuuucccggGcuc.....................    1      1    Mm2
.......................................................................aauugcacuuuucccggccuG.....................    1      1    Mm2
.......................................................................aauugcacuuuucccggUcuc.....................    4      1    Mm2
.......................................................................aauugcacuuuucccggccCc.....................    1      1    Mm2
.......................................................................aauugcacuuGucccggccuc.....................    1      1    Mm2
.......................................................................aUuugcacuuuucccggccuc.....................    1      1    Mm2
.......................................................................aauugcacuuuucccggAcuc.....................    4      1    Mm2
.......................................................................aauugcacuuuucccggccuA.....................    6      1    Mm2
.......................................................................aauugcacuuuucccgAccuc.....................    1      1    Mm2
.......................................................................aauugcacuuuucccggccuU.....................    13     1    Mm2
.......................................................................aauugcaAuuuucccggccucu....................    3      1    Mm2
.......................................................................aauugGacuuuucccggccucu....................    1      1    Mm2
.......................................................................aauugcacuuuuccUggccucu....................    15     1    Mm2
.......................................................................aauugcacuuuucccggccucC....................    25     1    Mm2
.......................................................................aauugcacuAuucccggccucu....................    1      1    Mm2
.......................................................................aauugcacuuuAcccggccucu....................    3      1    Mm2
.......................................................................aauugcUcuuuucccggccucu....................    3      1    Mm2
.......................................................................aauugcacuuuucccAgccucu....................    11     1    Mm2
.......................................................................aauugcacuuuuAccggccucu....................    2      1    Mm2
.......................................................................aauugcacuuuucUcggccucu....................    12     1    Mm2
.......................................................................aauugcacuuuucccggccucA....................    40     1    Mm2
.......................................................................aauugcNcuuuucccggccucu....................    4      1    Mm2
.......................................................................aauGgcacuuuucccggccucu....................    1      1    Mm2
.......................................................................aauugcacGuuucccggccucu....................    1      1    Mm2
.......................................................................aauugcacuuuucAcggccucu....................    10     1    Mm2
.......................................................................aauAgcacuuuucccggccucu....................    3      1    Mm2
.......................................................................aauuUcacuuuucccggccucu....................    8      1    Mm2
.......................................................................aauugcacuuuucccggUcucu....................    166    1    Mm2
.......................................................................Nauugcacuuuucccggccucu....................    15     1    Mm2
.......................................................................aaAugcacuuuucccggccucu....................    5      1    Mm2
.......................................................................Cauugcacuuuucccggccucu....................    4      1    Mm2
.......................................................................aauugcacuuuucccggccucu....................    32530  0    Mm2
.......................................................................aUuugcacuuuucccggccucu....................    6      1    Mm2
.......................................................................aauugcGcuuuucccggccucu....................    6      1    Mm2
.......................................................................aauugcacCuuucccggccucu....................    13     1    Mm2
.......................................................................aauugcacuuuucccggcUucu....................    6      1    Mm2
.......................................................................aaCugcacuuuucccggccucu....................    4      1    Mm2
.......................................................................aauugcaGuuuucccggccucu....................    1      1    Mm2
.......................................................................aauugcacuuCucccggccucu....................    25     1    Mm2
.......................................................................Gauugcacuuuucccggccucu....................    5      1    Mm2
.......................................................................aCuugcacuuuucccggccucu....................    4      1    Mm2
.......................................................................aauugcacuuuucccgUccucu....................    72     1    Mm2
.......................................................................aauugcaUuuuucccggccucu....................    3      1    Mm2
.......................................................................aauuCcacuuuucccggccucu....................    5      1    Mm2
.......................................................................aauugcacuuuGcccggccucu....................    1      1    Mm2
.......................................................................aauCgcacuuuucccggccucu....................    5      1    Mm2
.......................................................................aauugcacuuuucccggccuUu....................    12     1    Mm2
.......................................................................aauugNacuuuucccggccucu....................    2      1    Mm2
.......................................................................aauugcacuuuCcccggccucu....................    1      1    Mm2
.......................................................................aauugcacuuuucccgCccucu....................    91     1    Mm2
.......................................................................aauugcacuuuuUccggccucu....................    13     1    Mm2
.......................................................................aauugcacuuuucccUgccucu....................    3      1    Mm2
.......................................................................aauugUacuuuucccggccucu....................    12     1    Mm2
.......................................................................aauugcacuGuucccggccucu....................    4      1    Mm2
.......................................................................aauugcacuuuucccggccuAu....................    2      1    Mm2
.......................................................................aauuAcacuuuucccggccucu....................    5      1    Mm2
.......................................................................aauugcacuuAucccggccucu....................    65     1    Mm2
```

```
                                  Star                                Mature
accccaaacccuggaucucccggcugcuuagcaaaggcugugauuggugcaaaauucuguugucaacaagaauugcacuuuucccggccucugugcuguggccaguucuucc
......................................................................aauugcacuuuuccGggccucu....................    3      1     Mm2
......................................................................aauugcCcuuuucccggccucu....................    1      1     Mm2
......................................................................aauugcacuuuuccAggccucu....................    3      1     Mm2
......................................................................aauugcacuuuucccggGcucu....................    118    1     Mm2
......................................................................aauugcacuuuucGcggccucu....................    1      1     Mm2
......................................................................aauugcacuuuucccggcGucu....................    1      1     Mm2
......................................................................aauugcacuCuucccggccucu....................    6      1     Mm2
......................................................................aauugcacuuuucccgAccucu....................    87     1     Mm2
......................................................................aauugcacuuuucccggccuGu....................    113    1     Mm2
......................................................................aauugcacuuuucccggccCcu....................    2      1     Mm2
......................................................................aauugcacuuuucccggccucG....................    362    1     Mm2
......................................................................aaGugcacuuuucccggccucu....................    7      1     Mm2
......................................................................aauugcacuuGucccggccucu....................    50     1     Mm2
......................................................................aGuugcacuuuucccggccucu....................    8      1     Mm2
......................................................................Uauugcacuuuucccggccucu....................    37     1     Mm2
......................................................................aauugcacuuuucccggAcucu....................    288    1     Mm2
......................................................................aauugcacuuuucccggccucuU...................    1178   1     Mm2
......................................................................aauugcacuuuucccggccucug...................    9      0     Mm2
......................................................................aauugcacuuuucccggccucuA...................    119    1     Mm2
......................................................................aauugcacuuuucccggccucuC...................    3      1     Mm2
......................................................................aauugcacuuuucccggccucugu..................    1      0     Mm2
......................................................................aauugcacuuuucccggccucuUu..................    343    1     Mm2
......................................................................aauugcacuuuucccggccucuAu..................    97     1     Mm2
......................................................................aauugcacuuuucccggccucuCu..................    2      1     Mm2
......................................................................aauugcacuuuucccggccucuUug.................    1      1     Mm2
.......................................................................auugcacuuuucccggccu......................    5      0     Mm2
.......................................................................auugcacuuuucccggccG......................    1      1     Mm2
.......................................................................auuCcacuuuucccggccuc.....................    1      1     Mm2
.......................................................................auugcacuuuucccggcAucu....................    1      1     Mm2
.......................................................................auugcacuuuucccggcGucu....................    1      1     Mm2
.......................................................................auugcacuuuucccggccucu....................    79     0     Mm2
.......................................................................auugcacuuuucccggccuGu....................    5      1     Mm2
.......................................................................auugcacuuuCcccggccucu....................    1      1     Mm2
.......................................................................auugcacuuuucccggccucug...................    4      0     Mm2
.......................................................................auugcacuuuucccggccucuU...................    9      1     Mm2
.......................................................................auugcacuuuucccggccucuA...................    7      1     Mm2
.......................................................................auugcacuuuucccggccucuUu..................    3      1     Mm2
.......................................................................auugcacuuuucccggccucugu..................    5      0     Mm2
.......................................................................auugcacuuuucccggccucuAu..................    3      1     Mm2
.......................................................................auugcacuuuucccggccucuguU.................    1      1     Mm2
........................................................................uugcacuuuucccggccucugu..................    2      0     Mm2
.........................................................................ugcacuuGucccggccucu....................    1      1     Mm2
..........................................................................gcacuuuucccggccucu....................    2      0     Mm2

......................................................................aauugcacuuuucccggc........................    1      0     Fm3
......................................................................aauugcacuCuucccggcc.......................    1      1     Fm3
......................................................................aauugcacuuAucccggccu......................    1      1     Fm3
......................................................................aauugcacuuuucccggccu......................    136    0     Fm3
......................................................................Uauugcacuuuucccggccu......................    1      1     Fm3
......................................................................aauugcacuuuucccggAcu......................    1      1     Fm3
......................................................................aauugcacuuCucccggccu......................    1      1     Fm3
......................................................................aauugcacuuuucccggGcu......................    2      1     Fm3
......................................................................aauugcacuuuucccggccuG.....................    1      1     Fm3
......................................................................aauugcacuuuucccggccucu....................    20     0     Fm3
......................................................................Uauugcacuuuucccggccucu....................    1      1     Fm3
......................................................................aauugcacuuGucccggccucu....................    1      1     Fm3
......................................................................aauugcacuuuucccggccucA....................    1      1     Fm3
......................................................................aauugcacuuuucccggccuGu....................    11     1     Fm3
.......................................................................auugcacuuuucccggccuGu....................    4      1     Fm3

..................................aggcugugauuggugcaaaauucu......................................................    1      0     Mm3
.....................................................................Caauugcacuuuucccggccu......................    1      1     Mm3
.....................................................................gaauugcacuuuucccggccu......................    1      0     Mm3
.....................................................................Caauugcacuuuucccggccucu....................    5      1     Mm3
......................................................................aauugcacuuuucccggc........................    4      0     Mm3
......................................................................aauugcacuuuucccggU........................    1      1     Mm3
......................................................................aauugcacuCuucccggcc.......................    1      1     Mm3
......................................................................aauugcacuuuucccggcU.......................    2      1     Mm3
......................................................................aauugcacuuuucccggcc.......................    34     0     Mm3
......................................................................aauugcacuuuucccggcA.......................    1      1     Mm3
```

Star Mature

```
accccaaacccuggaucucccggcugcuuagcaaaggcugugauugguggcaaaauucuguugucaacaagaauugcacuuuucccggccucugugcuguggccaguucuucc
.......................................................................aauuCcacuuuucccggccu......................    2      1    Mm3
.......................................................................aauugcacuuuuccUggccu......................    1      1    Mm3
.......................................................................aauugcacuuuucccggGcu......................    6      1    Mm3
.......................................................................aauugcacuuuucccggccA......................    17     1    Mm3
.......................................................................aauugcacuuuucccgUccu......................    5      1    Mm3
.......................................................................aaCugcacuuuucccggccu......................    1      1    Mm3
.......................................................................Gauugcacuuuucccggccu......................    1      1    Mm3
.......................................................................aauCgcacuuuucccggccu......................    1      1    Mm3
.......................................................................aauuAcacuuuucccggccu......................    1      1    Mm3
.......................................................................aUuugcacuuuucccggccu......................    1      1    Mm3
.......................................................................aauugcacuuuucccgCccu......................    5      1    Mm3
.......................................................................aauugcacuuuucccgAccu......................    4      1    Mm3
.......................................................................aauugcacuuuuUccggccu......................    1      1    Mm3
.......................................................................aauugcacuuCucccggccu......................    2      1    Mm3
.......................................................................aauugcacuCuucccggccu......................    5      1    Mm3
.......................................................................aauugcacuuuucccggccG......................    46     1    Mm3
.......................................................................Nauugcacuuuucccggccu......................    3      1    Mm3
.......................................................................aaGugcacuuuucccggccu......................    1      1    Mm3
.......................................................................aauugcacuuAucccggccu......................    8      1    Mm3
.......................................................................aauugcacuuuucccAgccu......................    1      1    Mm3
.......................................................................aauugNacuuuucccggccu......................    1      1    Mm3
.......................................................................Uauugcacuuuucccggccu......................    3      1    Mm3
.......................................................................aauugcacuuuuGccggccu......................    1      1    Mm3
.......................................................................aauugcacuuuucccggccu......................    2182   0    Mm3
.......................................................................aauugcacuuuucccggccC......................    8      1    Mm3
.......................................................................aauugcacuuuucccggAcu......................    23     1    Mm3
.......................................................................aauugcacuuuucccggUcu......................    16     1    Mm3
.......................................................................aauugcacuuuucccggccCc.....................    2      1    Mm3
.......................................................................aauugcacuuuucccggccuU.....................    39     1    Mm3
.......................................................................aauugcacuuAucccggccuc.....................    1      1    Mm3
.......................................................................aauugcacuuuucccCgccuc.....................    1      1    Mm3
.......................................................................aauugcacuuuucccggccuG.....................    7      1    Mm3
.......................................................................aauugcacuuGucccggccuc.....................    1      1    Mm3
.......................................................................aauugcacuuuucccggAcuc.....................    2      1    Mm3
.......................................................................aauugcacuuuucccggccuc.....................    127    0    Mm3
.......................................................................aauugcacuuuucccggccCcu....................    1      1    Mm3
.......................................................................aauugcacuuuucccggccuUu....................    5      1    Mm3
.......................................................................aauCgcacuuuucccggccucu....................    2      1    Mm3
.......................................................................aauugcCcuuuucccggccucu....................    1      1    Mm3
.......................................................................aauugcacuuuucccggccuGu....................    115    1    Mm3
.......................................................................aauuAcacuuuucccggccucu....................    2      1    Mm3
.......................................................................aauugAacuuuucccggccucu....................    2      1    Mm3
.......................................................................aauugUacuuuucccggccucu....................    4      1    Mm3
.......................................................................aauugcacuuuucAcggccucu....................    5      1    Mm3
.......................................................................aauAgcacuuuucccggccucu....................    1      1    Mm3
.......................................................................aauugcacuuuucccUgccucu....................    2      1    Mm3
.......................................................................aCuugcacuuuucccggccucu....................    1      1    Mm3
.......................................................................aauugcacuuuucccggccuAu....................    3      1    Mm3
.......................................................................aauugcacuuGucccggccucu....................    42     1    Mm3
.......................................................................aauugcacuuuucccggccucA....................    13     1    Mm3
.......................................................................aauugcacuuuucccggAcucu....................    77     1    Mm3
.......................................................................Uauugcacuuuucccggccucu....................    11     1    Mm3
.......................................................................aauugcacuuuucccggGcucu....................    30     1    Mm3
.......................................................................aauugcacuuuucccgCccucu....................    19     1    Mm3
.......................................................................aGuugcacuuuucccggccucu....................    1      1    Mm3
.......................................................................aauugcacuuAucccggccucu....................    11     1    Mm3
.......................................................................aauugcacuuuucccAgccucu....................    3      1    Mm3
.......................................................................aauugcacuuuucccgUccucu....................    17     1    Mm3
.......................................................................aauugcacuuuuccAggccucu....................    1      1    Mm3
.......................................................................aauugcacuuuuUccggccucu....................    1      1    Mm3
.......................................................................aauugcacuuuucccggccucC....................    7      1    Mm3
.......................................................................aauugcacuGuucccggccucu....................    1      1    Mm3
.......................................................................aauugcaUuuuucccggccucu....................    1      1    Mm3
.......................................................................aauugcacuuuucccggUcucu....................    70     1    Mm3
.......................................................................Gauugcacuuuucccggccucu....................    1      1    Mm3
.......................................................................Nauugcacuuuucccggccucu....................    7      1    Mm3
.......................................................................aauugcacuuuCcccggccucu....................    3      1    Mm3
.......................................................................aaAugcacuuuucccggccucu....................    1      1    Mm3
.......................................................................aaCugcacuuuucccggccucu....................    2      1    Mm3
.......................................................................aauugcacuuuuccUggccucu....................    3      1    Mm3
```

```
                                  Star                                    Mature
accccaaacccuggaucucccggcugcuuagcaaaggcugugauuggugcaaaauucuguugucaacaagaauugcacuuuucccggccucugugcuguggccaguucuucc
......................................................................aauugcacuuuAcccggccucu....................   2      1   Mm3
......................................................................aauugcacuuuucUcggccucu....................   3      1   Mm3
......................................................................aauugcacuuuucccggccucu....................   9576   0   Mm3
......................................................................aauugcacuuuucccggccucG....................   90     1   Mm3
......................................................................aauugcUcuuuucccggccucu....................   2      1   Mm3
......................................................................aauGgcacuuuucccggccucu....................   1      1   Mm3
......................................................................aauugcacuuuucccgAccucu....................   24     1   Mm3
......................................................................aauugcacuuCucccggccucu....................   13     1   Mm3
......................................................................aauugcGcuuuucccggccucu....................   2      1   Mm3
......................................................................aauuUcacuuuucccggccucu....................   3      1   Mm3
......................................................................aUuugcacuuuucccggccucu....................   1      1   Mm3
......................................................................aauugcacuuuucccggccucuC...................   1      1   Mm3
......................................................................aauugcacuuuucccggccucuU...................   451    1   Mm3
......................................................................aauugcacuuuucccggccucuA...................   52     1   Mm3
......................................................................aauugcacuuuucccggccucuAu..................   41     1   Mm3
......................................................................aauugcacuuuucccggccucuUu..................   102    1   Mm3
.......................................................................auugcacuuuucccggccuc.....................   1      0   Mm3
.......................................................................auugcacuuuucccggcAucu....................   1      1   Mm3
.......................................................................auugcacuuuuUccggccucu....................   1      1   Mm3
.......................................................................auugcacuuuGcccggccucu....................   1      1   Mm3
.......................................................................auugcacuuuucccggccuGu....................   3      1   Mm3
.......................................................................auugcacuuuucccggccucu....................   27     0   Mm3
.......................................................................auugcacuuuucccggccucuC...................   1      1   Mm3
.......................................................................auugcacuuuucccggccucuA...................   2      1   Mm3
.......................................................................auugcacuuuucccggccucuU...................   5      1   Mm3
.......................................................................auugcacuuuucccggccucug...................   1      0   Mm3
.......................................................................auugcacuuuucccggccucuAu..................   3      1   Mm3
.......................................................................auugcacuuuucccggccucugA..................   1      1   Mm3
.......................................................................auugcacuuuucccggccucugu..................   5      0   Mm3
```
